# Chloroform and the Constant Current in Neuralgia

**Published:** 1888-04

**Authors:** 


### Chloroform and the Constant Current in Neuralgia.

Prof. Adamkiewicz says he has obtained marvellous results from the combined action of chloroform and the constant current in facial and other forms of neuralgia. The electrode is made of hollow charcoal into which the chloroform is introduced, and from which the current sends it into the tissues. That this power of penetration may be thus obtained is thought to be shown in the fact that when chloroform is colored with gentian violet and applied in the manner described to the ear of a rabbit, the tissue becomes dyed. In the experiments with the human subject, the writer notes, at the commencement, the triple action of the constant current, the chloroform and a coudition of cataphoresis foll lowed by a burning sensation and, finally, anæsthesia. Severa- remarkable cases of cure are cited. Anæsthesia was not obtained when the nerves were deep-seated, nor in sciatica.—*Progres Medical.*

